# How artificial intelligence and machine learning can help healthcare systems respond to COVID-19

**DOI:** 10.1007/s10994-020-05928-x

**Published:** 2020-12-09

**Authors:** Mihaela van der Schaar, Ahmed M. Alaa, Andres Floto, Alexander Gimson, Stefan Scholtes, Angela Wood, Eoin McKinney, Daniel Jarrett, Pietro Lio, Ari Ercole

**Affiliations:** 1 University of Cambridge, Cambridge, UK; 2 University of California, Los Angeles, USA; 3 Cambridge University Hospitals NHS Foundation Trust, Cambridge, UK

**Keywords:** Clinical decision support, Healthcare, COVID-19

## Abstract

The COVID-19 global pandemic is a threat not only to the health of millions of individuals, but also to the stability of infrastructure and economies around the world. The disease will inevitably place an overwhelming burden on healthcare systems that cannot be effectively dealt with by existing facilities or responses based on conventional approaches. We believe that a rigorous clinical and societal response can only be mounted by using intelligence derived from a variety of data sources to better utilize scarce healthcare resources, provide personalized patient management plans, inform policy, and expedite clinical trials. In this paper, we introduce five of the most important challenges in responding to COVID-19 and show how each of them can be addressed by recent developments in machine learning (ML) and artificial intelligence (AI). We argue that the integration of these techniques into local, national, and international healthcare systems will save lives, and propose specific methods by which implementation can happen swiftly and efficiently. We offer to extend these resources and knowledge to assist policymakers seeking to implement these techniques.

## Introduction

Both the UK and the international community have seen an unbelievable amount of pressure put on their social and healthcare infrastructure over the past months. AI and machine learning can use data to make objective and informed recommendations, and can help ensure that scarce resources are allocated as efficiently as possible. Doing so will save lives and can help reduce the burden on healthcare systems and professionals.

This paper goes into detail about specific practical challenges faced by healthcare systems, and how AI and machine learning can improve decision-making to ensure the best outcomes possible. While the paper is primarily focused on the UK national healthcare system, the challenges and methods highlighted in the paper apply to other countries. First, AI and machine learning can help us identify people who are at highest risk of being infected by the novel coronavirus. This can be done by integrating electronic health record data with a multitude of “big data” pertaining to human-to-human interactions (from cellular operators, traffic, airlines, social media, etc.). This will make allocation of resources like testing kits more efficient, as well as informing how we, as a society, respond to this crisis over time. AI and machine learning can also help us work out which infected patients are more likely to suffer more severely from COVID-19. We can provide more accurate patient risk scores that will help clinical professionals decide who needs urgent treatment (and resources), and when.

Secondly, COVID-19 symptoms and disease evolution vary widely from patient to patient in terms of severity and characteristics. A one-size-fits-all approach for treatment doesn’t work. We also are a long way off from mass-producing a vaccine. Machine learning techniques can help determine the most efficient course of treatment for each individual patient on the basis of observational data about previous patients, including their characteristics and treatments administered. We can use machine learning to answer key “what-if” questions about each patient, such as “What if we postpone a couple hours before putting them on a ventilator?” or “Would the outcome for this patient be better if we switched them from supportive care to an experimental treatment earlier?”

Third, we have seen a large variety of approaches taken by decision-makers when deciding on policies to respond to COVID-19. This is true from the individual level (i.e. practitioners) all the way up to the government level. For example, differences in triaging protocols used by medical institutions and practitioners could mean that two patients with similar profiles will end up receiving different types of treatment depending on where they happen to live. It is hard to get a clear sense of which decisions result in the best outcomes. In such a stressful situation, it is also hard for decision-makers to be aware of the outcomes of decisions being made by their counterparts elsewhere. Once again, data-driven AI and machine learning can provide objective and usable insights that far exceed the capabilities of existing methods. We can gain valuable insight into what the differences between policies are, why policies are different, which policies work better, and how to design and adopt improved policies. This information can be shared between decision-makers at all levels, improving consistency and efficiency across the board. The result is that routine decisions can be made in a more coordinated and timely way, freeing up valuable medical attention to the cases that demand real-time expertise.

Fourth, randomized clinical trials (RCTs) are generally used to judge the relative effectiveness of a new treatment. However, these trials can be slow and costly, and may fail to uncover specific subgroups for which a treatment may be most effective. A specific problem posed by COVID-19 is that subjects selected for RCTs tend not to be elderly, or to have other conditions; as we know, COVID-19 has a particularly severe impact on both those patient groups.

Rather than recruiting and assigning subjects at random, machine learning methods can recruit subjects from identifiable subgroups, and assign them to treatment or control groups in a way that speeds up learning. These methods have been shown to significantly reduce error and achieve a prescribed level of confidence in findings, while also requiring fewer subjects. We can also use machine learning to target particular treatments to specific subgroups and to understand what treatments are suitable for the population as a whole.

These four practical challenges form the key research avenues that we discuss in more detail in Sect. 2, whose focus is not to provide solutions but rather outline some of the key opportunities for the machine learning and AI researchers to contribute to the containment of the Covid-19 pandemic

In addition to the aforementioned practical challenges, the COVID-19 pandemic inspires various other research threads that are rather underdeveloped in existing literature. In particular, because we still know very little about the COVID-19 pandemic, and the virus itself may continue to change over time, we may not be able to rely on the data from decisions and outcomes taken in other countries, as those may generalize poorly to different countries. In the meantime, unproven hypotheses about the disease are likely to propagate online, impacting individual behaviour and causing systemic risks. This encourages more progress in an area of machine learning called transfer learning to account for differences between populations, substantially eliminating bias while still extracting usable data that can be applied from one population to another. In addition, we need methods to make us aware of the degree of uncertainty of any given conclusion or recommendation generated from machine learning. This means that decision-makers can be provided with confidence estimates that tell them how confident they can be about a recommended course of action. We discuss these research challenges in Sect. 3.

### Practical challenges

#### Managing limited healthcare resources

Healthcare systems in the UK and elsewhere in the world will soon face a severe scarcity of resources—in particular testing kits, hospital beds, ICU beds, ventilators and personnel. The UK, for example, currently has capacity for only a limited amount of tests to be conducted per day (https://www.wired.co.uk/article/coronavirus-testing-uk) and many of the intensive care unit (ICU) beds are already occupied (https://www.independent.co.uk/news/health/coronavirus-europe-intensive-care-beds-ventilatorsa9403981.html). This scarcity of resources is aggravated by the fact that individuals with COVID19 are known to experience widely different patterns of disease progression and outcomes: while some patients are asymptomatic, others manifest flu-like symptoms of varying severity, and some experience complications such as pneumonia and fatal multi-organ failure (Jiang et al. 2020). Since resources are limited and risk and disease progression so heterogeneous, it is crucial to identify—as early as possible—*which individuals* are likely to have been infected by the virus, *which infected individuals* may experience adverse events, *which types of medical resources* those individuals will require, and when these resources will be required.

The scarcity of healthcare resources will be exacerbated by the need to employ those resources to deal with both COVID-19 cases and with other patients who require—or will require—medical care. There is thus a clear and urgent need to deploy systems that can provide early warnings for personalized risk and disease progression of individuals, and that can inform medical personnel and healthcare systems about which patients would benefit from what resources and when (https://www.nytimes.com/2020/03/21/us/coronavirus-medical-rationing.html).

**How AI can help**

Mature AI-based support systems for a number of chronic diseases already exist, but a pandemic such as COVID19 presents a problem of a different nature. While chronic diseases jeopardize *individual health*, pandemics jeopardize *public health* because they present both *individual* and *systemic* risks. Pandemics, by nature, undermine the underlying social structures that connect individuals together. Dealing with these systemic risks requires merging clinical data with a variety of diverse social data. Electronic health records (EHR) hold data that can be used to pinpoint individuals’ clinical risk factors (Wang et al. 2020), and which can be linked to the multitude of “big data” pertaining to human-to-human interactions (e.g. data from cellular operators, traffic, airlines, and social media). Machine learning (ML) is especially suited for merging these various sources of data to issue accurate predictions of risk and help uncover the social structures through which systemic risks manifest and spread.

One of the strengths of ML is its ability to “learn” how an individual’s features (risk factors), including clinical and social information, can be mapped into *personalized predictions of risk* (Alaa and van der Schaar 2018). While standard epidemiological approaches—such as the Cox proportional hazards model—are unable to effectively combine data from diverse data sources and modalities (e.g. demographic, social, longitudinal, imaging, multi-omics), modern techniques for representation learning based on neural networks can readily and effectively learn from such diverse data to issue personalized predictions of risk as well as to update these predictions as features evolve over time. For instance, learning can be informed by dynamic models of social interaction (Alaa et al. 2018; Xu et al. 2014), and hence can predict the likelihood that an individual has been, or will be, in contact with a coronavirus carrier. This makes it possible to better allocate testing kits such that individuals most likely to have been exposed to the virus can be identified and tested most promptly. Such risk prediction mechanisms can be used for disease prevention, monitoring and detection.

With regard to individuals who have already been infected with SARS-CoV-2, it is important to be able to predict, on a personalized basis, the risk of experiencing adverse events (including mortality) as well as the specific healthcare resources a patient will/might need, and the timing of these needs. Key factors determining risk include patient age and/or the existence of comorbidities, such as hypertension, cystic fibrosis, or immunosuppression associated with transplants and chemotherapy for cancer (Zhou et al. 2020). However, the current risk-scoring methods for infected patients employ only a few factors such as Clinical Frailty Score, the number of comorbidities, or use of medication or administrative data from previous hospital admissions, to predict which patients are at highest risk. These relatively crude approaches (such as using the Charlson score (Goldstein et al. 2004)) may be appropriate in the frenetic acute stages of this epidemic, but they fail to capture and account for the subtle interactions between age, specific comorbidities (including their duration), organ failure scores and the impact of the wider determinants of health. Moreover, reliance on such measures may also result in failure to discover new risk factors—which may be especially important for a disease about which so little is known. Methods based on automated ML (Alaa and van der Schaar 2018; Lee et al. 2019; Zhang et al. 2020) can address both of these failings, offering increasingly accurate subtypings of the COVID-19 disease as more data is gathered, while simultaneously uncovering new risk factors as well as interactions among new and established risk factors.

Moreover, patients with severe comorbidities may experience diverse adverse events, especially because their usual treatments may be suspended in order to avoid compromising their immune response. In these cases, ML methods for assessing competing risks at the individual level– estimating the probabilities of a patient experiencing different adverse events and how these probabilities are changing over time—have shown improved prediction accuracy compared to conventional epidemiological methods ( Alaa and van der Schaar 2018). These methods can play an essential role in guiding decisions for individual patients.

As noted, patients diagnosed with COVID-19 experience a wide variety of disease trajectories and outcomes, such as heterogeneous incubation periods and variable rates of in-hospital pulmonary decline (Jiang et al. 2020). As a consequence, risk assessment must involve more than just a single time point prediction of final outcomes, and must also take account of the evolution of disease severity. This provides not only a way to predict a patient’s disease trajectory but—by including follow up data—it enables understanding of how specific events (e.g. symptoms worsening) may move patients from one trajectory or cluster/temporal phenotype to another. This is fundamental to the delivery of systems that allow the dynamic management of limited healthcare resources.

There are two routes by which resource allocation should be an active choice guided by AI-based risk assessment:

(a) *Patient-level* resource allocation concerning decisions made on an individual basis. This includes such decisions as who is most likely to benefit from early admission to an ICU, which patients can be safely stepped down from intensive support, or which patients can be safely discharged from the hospital and when.

Such questions of timely admission and discharge are often answered on the basis of simple rules of thumb (e.g. examining the most recent measurements for a select few variables, and making decisions based on threshold values). A more tailored approach to triaging should involve incorporating information from the individual patient’s history using all available observations at any point in time. Such triaging systems should be informed by state-of-the-art AI-based early warning systems trained on temporal (longitudinal) data. These AI-enabled systems have been developed and tested, and have shown their efficacy in practice (Yoon et al. 2016; Lee et al. 2018). They can anticipate a patient’s future healthcare needs, and can also handle irregularly collected data and impute missing features (Yoon et al. 2018a, b), as well as inferring important predictors of patient outcomes (e.g. specific comorbidities that are more likely to worsen the prognosis of a COVID-19 patient) (Yoon et al. 2018; Jordon et al. 2018). Importantly, ML and AI approaches are well-suited for handling the irregularly-sampled temporal data common in this setting, employing mixtures of Gaussian processes (Alaa et al. 2017), recurrent neural networks (Lee et al. 2019), state-space models (Alaa et al. 2019), neural network-based point processes (Qian et al. 2020), Bayesian non-parametric approaches (Bellot and Schaar 2020).

(b) *Measurement-level* resource allocation (value of information). Making observations and collecting healthcare data is costly in terms of time, materials, and personnel—and not all data is equally valuable. We need to actively decide what we should measure, when it should be measured, and on which patients; making observations on each patient should be an active and informed process, so that the information gained can be most valuable. Developments in the field of active sensing offer tools for optimally trading off the value of information against the cost of acquisition (Yoon et al. 2019, 2018).

Taken together, techniques in patient- and measurement-level prediction and active sensing can integrate information across time in order to effectively allocate resources among patients while prioritizing measurements and treatments, thereby enabling institutions to dynamically allocate hospital resources depending on their patients’ needs. This, in turn, allows physicians to make informed decisions to postpone treatment for conditions that are not immediately life threatening, so that resources can be used for critically-ill COVID-19 patients.

### Developing personalized patient management and treatment plans

Vaccines and therapeutics for COVID-19 will take some time to be developed and introduced (Lu 2020). Although a number of antiviral medications are being trialed, none are currently known to be effective. Due to the heterogeneous nature of patient disease trajectories, one-size-fits-all treatment plans (which are developed at the population level, rather than individualized patient level) are very likely to be ineffective in terms of both patient outcomes and use of limited resources. To save lives and effectively use scarce resources, it is therefore essential to develop management and treatment plans that are personalized for each patient, depending on their unique characteristics and needs.

**How AI can help**

AI-based models can efficiently use available observational data to learn the effects of existing treatments on individuals, given their specific features (characteristics) including existing comorbidities. This can help identify the best management approach for each patient based on their features, including the best policies for employing mechanical ventilation and/or experimental treatments.

Learning personalized treatment *effects* can be carried out using recent advances in machine learning models tailored for learning in the observational data setting. Models based on Gaussian processes (Ahmed et al. 2017), generative adversarial networks (Yoon et al. 2018), and deep neural networks (Zhang et al. 2020) can learn individual-level effects of experimental treatments on the basis of observational data (without resorting to randomized trials), and can potentially be integrated with omics data. Using such methods, we can identify patient subgroups for whom treatments such as Remdesivir, Lopinavir/Ritonavir and Chloroquine may be effective (Gao et al. 2020).

Learning personalized *management and treatment plans* requires modeling the evolution of a patient’s health state as they respond to different sequences of treatments and supportive care over time during hospitalization. AI-based models (Ahmed et al. 2017; Yoon et al. 2018; Zhang et al. 2020) can answer what-if questions such as: “what would happen to a given patient if mechanical ventilation were delayed for a few hours?” or “what would a given patient’s outcomes be if we switched from supportive care to a specific experimental treatment a week earlier?”

Because each patient is different, answering *counterfactual* questions such as these requires efficient use of regularly-updated observational data from patients who have experienced different management plans since the beginning of the viral outbreak; this can help us learn what the best plan is for each individual given the available healthcare resources. The counterfactual questions under consideration here pertain not only to *which* intervention should be applied for each patient, but also *when* the patient needs this intervention. This is especially relevant given the limited resources available.

Leveraging longitudinal patient observational data, the counterfactual recurrent network in Bica et al. (2020) learns to predict counterfactual health trajectories under different management plans for each individual. Such predictions can be made in a dynamic fashion by modeling and updating the patient’s state over time to account for information in all past medical events. Doing this can pinpoint a timeframe during which intervention would be most effective, as well as choosing the specific sequence of interventions that would result in the best outcome for each patient. In addition, this method has the potential to show when scarce interventions—such as the mechanical ventilator—are not critically needed by a patient, and for how long their use can be postponed without the patient suffering significant deterioration.

### Informing policies and enabling effective collaboration

Governmental and local responses to the outbreak have been remarkably varied (Ferguson et al. 2020). At the national, regional, institutional and even individual practitioner level, very different decisions are being made in terms of prevention, diagnosis, treatment and disease management. In uncertain settings—like the ones presented by COVID-19—policymakers and clinical practitioners make different observations, take different measurements, and act differently on the basis of evidence. For example, differences in triaging protocols across institutions and practitioners could mean that two patients with similar profiles end up receiving different courses of treatment simply because of where they happen to be seen.

There is little consensus as to what policies and decisions are most effective; as a result, there is substantial variation in efficiency of resource usage, quality of care, and patient outcomes. It is imperative that we employ systematic, data-driven methods in learning from the policies already implemented in the wild. Coordinating experiences across different actors allows us to more systematically quantify policies and their efficacies, thereby informing the design of improvements.

**How AI can help**

Evidence-based decision-making entails collecting costly observations about the effects of a treatment or resource allocation policy, and subsequently committing to an informed decision on the basis of accumulated observations. AI methods tackle the following important and interrelated questions, and offer a common language through which healthcare practitioners can exchange information and improve policies over time.

*What are the different diagnostic and treatment policies?*

First, we can quantify the *decision rules* that underlie observed behavior (e.g. the guidelines and protocols often implicitly encoded in different institutions). For a concise, standardized *description* of behavior, what is learned here is an accessible mapping from information (e.g. patient characteristics and measurements) to actions (i.e. diagnostic tests and treatments)—a mapping that is immediately indicative of which individual actions are more or less likely to be taken in any given scenario. For instance, precisely how badly does a patient have to be doing before a ventilator is employed? How does this compare across hospitals? Modeling policies transparently enables identification and comparison of actions by different decision-makers.

*What are the priorities demonstrated by different policies?*

Second, we can quantify the *preferences* and potential biases implicit in observed behavior (e.g. what treatments are preferred *ceteris paribus*, and how time-sensitivity and monetary costs weigh on decisions). For a concise, standardized *interpretation* of different policies, what is obtained is an accessible mapping from behavior to preferences—a mapping that is immediately indicative of what aspects of the decision-making process (e.g. expense, accuracy, time pressure, and patient populations) appear more or less important for a given decisionmaker. For instance, we may expect that an institution diagnoses and treats older patients more quickly than younger ones. But do they actually? And if so, by how much?

*What are the best improvements to existing policies?*

Third, we can quantify the *efficacy* of both new and existing policies, so as to identify the best policy to implement under different priorities and in different circumstances. While answering the two directly preceding questions can facilitate transparent comparison and understanding of variations in clinical practice, here the culminating objective is in the *prescription* of better diagnosis and treatment policies. This can be accomplished by integrating the results of answering both of the preceding questions with novel techniques in reinforcement learning to inform and instruct policy evaluation and policy improvement. For instance, knowing that we desire to optimize for a certain agreed-upon set of priorities, precisely what is the best decision rule to implement going forward?

*What is the level of confidence in learned policies?*

Finally, we can quantify the *uncertainty* involved in these tasks of identifying, understanding, and improving decisions and policies. This is critical in new environments where existing predictive expertise is lacking—such as in responding to COVID-19. For instance, novel diagnostic tests will certainly have errors, and the overall effectiveness of different treatments is not known *a priori*. Quantifying uncertainty permits a clear understanding of which components of our models we can have more confidence in, and which components we cannot. For instance, when can we confidently assert that a given action is necessary, and when does the choice require more nuanced deliberation?

In the presence of constraints on medical personnel, the potential for decision support through policy evaluation, improvement, and well-calibrated uncertainty estimates enables routine decisions to be taken in a more timely manner—thus freeing up valuable medical attention to the cases that warrant real-time expertise. Recent work in tackling some of these challenges involves quantifying and explaining behavior in the context of early diagnosis (Hüyük and Jarrett 2020), modeling and understanding sequential decisions under uncertain deadline pressure (Jarrett 2020), and learning interpretable parameterizations of behavior via counterfactual reasoning to highlight cost benefit tradeoffs associated with different actions over time (Bica et al. 2020). There is, therefore, enormous opportunity for AI to facilitate healthcare professionals in making better decisions by learning from the “experimentation” currently taking place in different settings for different patients.

### Expediting clinical trials

In order to compare the effectiveness of a new treatment to an existing one (or to a placebo), randomized clinical trials (RCTs) are considered the gold standard. However, it is a well-known fact that RCTs can be slow and costly, and may fail to uncover specific sub-populations for which a treatment would be most effective. Moreover, conclusions drawn from RCTs are typically valid only for the types of patients recruited for that RCT. This is an especially significant problem in the case of COVID-19, as elderly patients and patients with comorbidities, who are known to be at higher risk, are typically excluded from RCTs.

**How AI can help**

Recent work on improving the design of adaptive clinical trials has demonstrated that both the efficiency and effectiveness of RCTs can be significantly enhanced through the application of ML (Zame et al. 2020).

While most RCTs simply allocate patients to treatment and control groups through uniform randomization, this procedure can be highly sub-optimal in terms of learning. ML-enabled adaptive clinical trials (Lee et al. 2020; Atan et al. 2019; Zame et al. 2020) can recruit patients in cohorts (rather than all at once), the effects on each cohort can be observed before recruiting the next cohort, and may be heterogeneous across identifiable subgroups of patients (as would be expected for COVID-19 treatments). Rather than recruiting and assigning subjects at random, ML methods can recruit subjects from identifiable subgroups, and assign subjects to treatment or control groups in a way that speeds up learning. These methods have been shown to significantly reduce error and achieve a prescribed level of confidence in findings, while requiring many fewer patients.

Another important application of ML in the domain of RCTs is the post-hoc analysis of clinical trials, and, in particular, the identification of sub-populations that are most similar in characteristics (array of features) and treatment response. The identification of such sub populations is informative in itself because it permits inference about the extent of heterogeneity and response across the entire population. Moreover, quantifying the uncertainty associated with the treatment of specific sub-populations makes it easier to target particular treatments to those sub-populations and understand what treatments are suitable for the population as a whole.

## Research challenges: accounting for uncertainty

Because it is novel, the COVID-19 pandemic is being managed with very limited prior experience and limited data. Our knowledge of its behavior is very uncertain (Anderson et al. 2020). Moreover, since the coronavirus may undergo further natural selection and evolve geospatially as the pandemic progresses, understanding this evolution and the uncertainties that accompany it will be key to mitigation.

To work effectively within this environment, we need to quantify the *degree of uncertainty* in our knowledge about the disease, and then apply this to understand the risks and benefits of the clinical and social policies employed to mitigate it. This is especially important because unproven hypotheses about COVID-19 have already circulated rapidly online and through various media outlets, and are likely to impact individual behavior and hence affect systemic risks.

There are three particular sources of uncertainty encountered when dealing with the current COVID-19 pandemic. First, models trained using data from the population tested for SARS-CoV-2 may be biased, since patients who have been tested are not necessarily representative of the overall UK population. Relying on data from other countries (e.g. China, Italy, Iran or South Korea) where the outbreak took off earlier may lead to biased models that do not generalize well to the UK population. Second, there is a limited amount of data available about COVID-19 patients. In light of the vast heterogeneity of patient characteristics and outcomes  (Alaa et al. 2020), blanket hypothesis tests about the disease may appear erroneously inconclusive. Finally, since risk predictors and indicators of COVID-19 outcomes are unknown, there is a great deal of uncertainty regarding the value of information conveyed in different national and patient-level factors.

**How AI can help**

ML techniques provide a wide variety of solutions for modeling uncertainty in these three settings:

The problem of biased models can be addressed via *transfer learning* methods, which implicitly cope with uncertainty in predictions made for individuals from populations other than those used for training. International collaboration could produce a large dataset comprising data from multiple infected populations, and a single two-level risk assessment model can be trained on such a dataset and deployed worldwide. However, since the characteristics of the UK population differ from other populations (e.g. the median age in the UK is 40.5 years, whereas for Iran it is 31.3 years), the model needs to “learn” its uncertainty in predictions made for specific individuals in the UK. Transfer learning models, e.g., based on transductvie dropout (Chan et al. 2020), accomplish this task effectively, and have been previously shown to work well for prognostication of disease across different countries or healthcare systems.

Transfer learning is relevant not only for transferring models *across populations*, but also for updating ML models for a single population as the diagnosed cohort evolves *over time*. Specifically, as diseases and populations evolve over time, observed distributions of patients—and their characteristics and outcomes—may change. Using successive batches of data as they arrive, scalable Bayesian optimization techniques have been developed to update models with new information for continued performance, while efficiently leveraging past optimizations (Zhang et al. 2019).

Importantly, we need to know what we *do* know and what we *do not* know when providing decision-makers with actionable information based on AI-systems. For this, new technologies have been developed to systematically quantify uncertainty in the predictions made by machine learning models (Ahmed 2020; Alaa and van der Schaar 2020). We need to mandate that all predictions generated by AI-enabled systems for decision support also come with confidence estimates. Decision-makers will then know when and for which patients they can trust the predictions issued by the AI system, and when they cannot have the same degree of certainty.

Quantifying uncertainty on predictions of patient-level outcomes is crucial for guiding hospitalization and therapeutic decisions. It is also important that uncertainty measures provide guarantees on their coverage performance, i.e., the likelihood that a confidence interval covers the true outcome. Recently proposed methods based on *influence functions* enables obtaining *Jackknife* estimates of machine learning outcomes in a *post-hoc* fashion, i.e., it does not depend on the model architecture or design choices. These methods have been applied to both models of cross-sectional and longitudinal data (Ahmed 2020; Alaa and van der Schaar 2020).

Active sensing is another key area where uncertainty quantification can play a crucical role: by quantifying the reduction of uncertainty in patient-level predictions associated with different types of information, we can decide on *which information to gather* in order to build data sets that would further be used to train machine learning models. This can be achieved using recently proposed actor-critic methods for active sensing and estimation of value of information (Yoon et al. 2019, 2018).

## Recommended means of implementing the techniques

For the variety of AI-based models discussed, deployment can take advantage of existing data infrastructure. Information about patients from EHR records, can be combined—through collaboration between public and private sectors—with data from cellular operators, airlines, traffic applications, and social media. Such collections of static and temporal data streams can be integrated by AI models to enable more personalized predictions of risk and treatment effects, patient management policies, and better trial design.

To illustrate what is possible, we will consider the UK. Countries with already existing virus surveillance systems, such as the UK, are in an excellent position to capitalize on the available data and employ the aforementioned AI technologies to improve outcomes. Recent emergency privacy legislation has allowed the collection, linkage and analysis of record-level, patient-identifiable data by the national health and social care data aggregator, NHS Digital. In addition, all COVID-19 testing data is collated nationally by Public Health England, while patients suspected to have been infected are also triaged through a national health triage service, with the data from calls being made available in real time. Daily service-level reporting of resource utilization across the acute sector is being expanded, while similar capacity monitoring is being introduced across social care. Taken together, such nationally-collated datasets provide a fertile environment to implement the techniques outlined in this paper.

Implementation of proposed AI-based methods can be accomplished through three levels of human-machine interface:

(a) *Individual-level interface.* Based on individual health risk assessments issued by AI models, patients can be alerted to undergo testing via email, mobile phone calls, and text messages. GPS-powered mobile applications installed by individuals on their cell phones can be used to guide social distancing by notifying individuals in high-risk geographical locations where a significant number of COVID-19 cases have been diagnosed, or where a significant number of high risk potential carriers of the virus are currently present. Implementation of this system can be primarily based on software applications that can be promptly executed and linked to NHS-held GP patient registries.

(b) *Hospital-level interface.* For public health risk assessments, existing in-hospital EHR infrastructure can be complemented with risk-modeling applications that display and alert clinicians to the evolving risks of patients within each hospital. Existing telehealth applications adopted by the NHS’s Technology Enabled Care Services (TECS) program can be utilized for distant diagnosis and care for self-isolating individuals with evident symptoms. Implementation of AI-based models in hospitals can largely rely on existing hardware infrastructure to link input data streams from ICUs to software (powered by ML models) that displays patient risks to clinicians.

(c) *Nation-level interface.* Government officials and decision-makers can be supported with centralized applications that continuously update officials with the most recent epidemiological data on COVID-19, in addition to geographically-stratified aggregate risk assessments and hospital occupancy rates for the entire population. This can be directly linked to the NHS patient registration database, and would interface with the other applications to collect information on population risk. This can help officials make selective strategic decisions on which locations to lock down, and which hospitals need more resources.

**Fig. 1:**
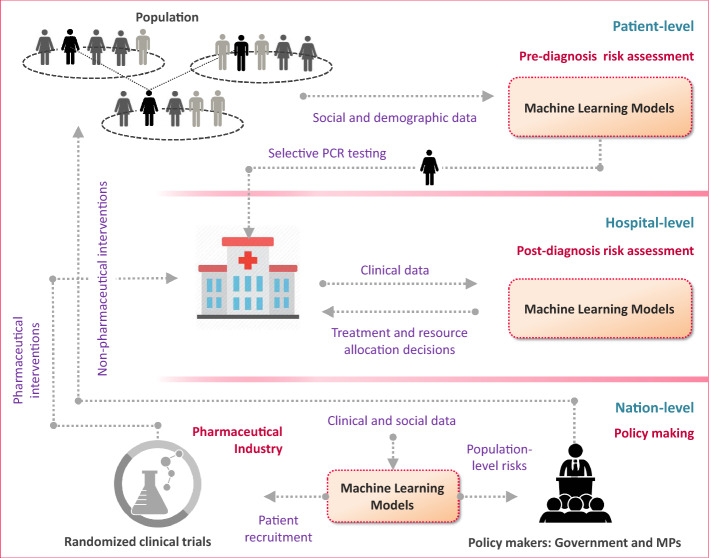
High-level illustration of how the proposed ML and AI solutions can help healthcare systems respond to COVID-19

In the UK, the National Health Service provides a centralized route to deployment for a whole country, while for example in the US, implementation may be most effective through large provider networks, HMOs or insurers. Figure 1 provides a high-level illustration of how the proposed ML and AI solutions can help healthcare systems respond to COVID-19.

We hope that a variety of safe and trustworthy machine learning methodologies will be developed and implemented worldwide to assist users (policymakers, clinicians, patients, etc.) in their decision-making processes.

## References

[CR1] Ahmed, M. A., & van der Schaar, M. (2017). Bayesian inference of individualized treatment effects using multi-task gaussian processes. *NeurIPS* 2017.

[CR2] Ahmed, M. A., & van der Schaar, M. (2020). Discriminative Jackknife: Quantifying uncertainty in deep learning via higher order influence functions.

[CR3] Alaa, A. M., & van der Schaar, M. (2018). AutoPrognosis: Automated clinical prognostic modeling via Bayesian optimization with structured kernel learning.

[CR4] Alaa, A. M., & van der Schaar, M. (2019). Attentive state-space modeling of disease progression. *Advances in Neural Information Processing Systems*. 2019.

[CR5] Alaa, A. M., & van der Schaar, M. (2020). Frequentist uncertainty in recurrent neural networks via blockwise influence functions.

[CR6] Alaa, A. M, Yoon, J., Hu, S., & van der Schaar, M. (2017). Personalized risk scoring for critical care prognosis using mixtures of gaussian processes. *IEEE Transactions on Biomedical Engineering*, 2017. 10.1109/TBME.2017.2698602 28463183

[CR7] Alaa AM, Ahuja K, van der Schaar M (2018). A micro-foundation of social capital in evolving social networks. IEEE Transactions on Network Science and Engineering.

[CR8] Anderson RM, Hans H, Don K, Hollingsworth TD (2020). How will countrybased mitigation measures influence the course of the COVID-19 epidemic?. The Lancet.

[CR9] Atan, O., Zame, W. R., & van der Schaar, M. (2019). Sequential patient recruitment and allocation for adaptive clinical trials. In *International Conference on Artificial Intelligence and Statistics (AISTATS)*, 2019.

[CR11] Bellot, A., & van der Schaar, M. (2020). Flexible modelling of longitudinal medical data: A Bayesian nonparametric approach. *ACM Transactions on Computing for Healthcare*, 2020.

[CR12] Bica, I., Alaa, A. M., Jordon, J., & van der Schaar, M. (2020). Estimating counterfactual treatment outcomes over time through adversarially balanced representations.

[CR13] Bica, I., Jarrett, D., Hüyük, A., & van der Schaar, M. (2020). Batch inverse reinforcement learning using counterfactuals for understanding decision making.

[CR14] Chan, A., Alaa, A. M., & van der Schaar, M. (2020). Unlabelled data improves bayesian uncertainty calibration under covariate shift.

[CR15] Ferguson, N. M., Daniel, L., Gemma, N. -G., Natsuko, I., Kylie, A., Marc, B., Sangeeta, B. et al. (2020). Impact of non-pharmaceutical interventions (NPIs) to reduce COVID-19 mortality and healthcare demand. London: Imperial College COVID-19 Response Team, March 16.

[CR17] Gao, J., Zhenxue, T., & Xu, Y. (2020). Breakthrough: Chloroquine phosphate has shown apparent efficacy in treatment of COVID-19 associated pneumonia in clinical studies. *BioScience Trends* (2020). 10.5582/bst.2020.01047 32074550

[CR18] Goldstein Larry B, Samsa Gregory P, Matchar David B, Horner Ronnie D (2004). Charlson Index comorbidity adjustment for ischemic stroke outcome studies. Stroke.

[CR23] Hüyük, A., Jarrett, D., van der Schaar, M. (2020). Explaining by Imitating: Understanding Decisions by Direct Policy Learning. Preprint.

[CR24] Jarrett, D., & van der Schaar, M. (2020). Inverse active sensing: Modeling and understanding timely decision-making. Preprint.

[CR25] Jiang F, Liehua D, Liangqing Z, Yin C, Chi WC, Zhengyuan X (2020). Review of the clinical characteristics of coronavirus disease (COVID-19). Journal of General Internal Medicine.

[CR26] Jordon, J., Jinsung Y., van der Schaar, M. (2018). KnockoffGAN: Generating knockoffs for feature selection using generative adversarial networks. In *International Conference on Learning Representations (ICLR)* 2018.

[CR28] Lee, H. -S., Shen, C., Jordon, J., van der Schaar, M. (2020). Contextual constrained learning for dose-finding clinical trials. In *International Conference on Artificial Intelligence and Statistics (AISTATS)*, 2020.

[CR29] Lee, C., William, R. Z., Jinsung, Y., & van der Schaar, M. (2018). Deephit: A deep learning approach to survival analysis with competing risks. In *Thirty-Second AAAI Conference on Artificial Intelligence (AAAI)*. 2018.

[CR30] Lee, C., Yoon, J., & van der Schaar, M. (2019). Dynamic-DeepHit: A deep learning approach for dynamic survival analysis with competing risks based on longitudinal data. *IEEE Transactions on Biomedical Engineering (TBME)*, 2019. 10.1109/TBME.2019.2909027 30951460

[CR31] Lee, C., Zame, W. R., Alaa, A. M., & van der Schaar, M. (2019). Temporal quilting for survival analysis. In *International Conference on Artificial Intelligence and Statistics (AISTATS)*, 2019.

[CR32] Lu H (2020). Drug treatment options for the 2019-new coronavirus (2019-nCoV). Biosci Trends.

[CR34] Qian, Z., Ahmed M. A., Alexis, B., Jem, R., & van der Schaar, M. (2020). Learning dynamic and personalized comorbidity networks from event data using deep diffusion processes. In *International Conference on Artificial Intelligence and Statistics (AISTATS)*, 2020.

[CR35] Qian, Z., Alaa, A. M., van Der Schaar, M. & Ercole, A. (2020). Between-centre differences for COVID-19 ICU mortality from early data in England. *Intensive Care Medicine (ICM)*, 2020. 10.1007/s00134-020-06150-y PMC7306496 32572526

[CR37] Wang, M., Qianyi, Z., Wenjun, C., Jeff, P., Honghan, W., Cathie, S., & Dave, R. (2020). Building the Knowledge Graph for UK Health Data Science. *ERA*.

[CR38] Xu J, van der Schaar M, Liu J, Li H (2014). Forecasting popularity of videos using social media. IEEE Journal of Selected Topics in Signal Processing (JSTSP).

[CR39] Yoon, J., Ahmed, A., Scott, H., & Mihaela, S. (2016). ForecastICU: a prognostic decision support system for timely prediction of intensive care unit admission. In *International Conference on Machine Learning* (pp. 1680–1689).

[CR40] Yoon, J., James, J. & van der Schaar, M. (2018). NVASE: Instance-wise variable selection using neural networks. In *International Conference on Learning Representations (ICLR)*, 2018.

[CR41] Yoon, J., James, J., & van der Schaar, M. (2018). GANITE: Estimation of individualized treatment effects using generative adversarial nets. In *International Conference on Learning Representations (ICLR)* 2018.

[CR42] Yoon, J., James, J., & van der Schaar, M. (2019). ASAC: Active sensing using Actor-Critic models. *Machine Learning for Healthcare* 2019.

[CR43] Yoon, J., Jordon, J. & Van Der Schaar, M. (2018). Gain: Missing data imputation using generative adversarial nets.

[CR44] Yoon, J., William, R. Z., & van der Schaar, M. (2018). Deep sensing: Active sensing using multidirectional recurrent neural networks. In *International Conference on Learning Representations (ICLR)* 2018.

[CR45] Yoon J, William RZ, van der Schaar M (2018). Estimating missing data in temporal data streams using multi-directional recurrent neural networks. IEEE Transactions on Biomedical Engineering.

[CR46] Zame, W. R., Bica, I., Shen, C., Curth, A., Lee, H. -S., Bailey, S., Weatherall, J., Wright, D., Bretz, F., van der Schaar, M. (2020). Machine learning for clinical trials in the era of COVID-19. Statistics in Biopharmaceutical Research—Special Issue on Covid-19, 2020. 10.1080/19466315.2020.1797867 PMC8011491 34191983

[CR47] Zhang, Y., Alexis, B., & van der Schaar, M. (2020). Learning overlapping representations for the estimation of individualized treatment effects. In *International Conference on Artificial Intelligence and Statistics (AISTATS)*, 2020.

[CR48] Zhang, Y., Jarrett, D., & van der Schaar, M. (2020). Stepwise model selection for sequence prediction via deep kernel learning. In *International Conference on Artificial Intelligence and Statistics (AISTATS)*, 2020.

[CR49] Zhang, Y., Jordon, J., Alaa, A. M., & van der Schaar, M. (2019). Lifelong Bayesian Optimization. arXiv preprint, 2019.

[CR50] Zhou, F., Ting, Y., Ronghui, D., Guohui, F., Ying, L., Zhibo, L., & Jie, X. et al. (2020). Clinical course and risk factors for mortality of adult inpatients with COVID-19 in Wuhan, China: A retrospective cohort study. *The Lancet*. 10.1016/S0140-6736(20)30566-3 PMC7270627 32171076

